# Forecasting COVID-19 in Pakistan

**DOI:** 10.1371/journal.pone.0242762

**Published:** 2020-11-30

**Authors:** Muhammad Ali, Dost Muhammad Khan, Muhammad Aamir, Umair Khalil, Zardad Khan

**Affiliations:** Department of Statistics, Abdul Wali Khan University Mardan, Mardan, KP, Pakistan; South China University of Technology, CHINA

## Abstract

**Objectives:**

Forecasting epidemics like COVID-19 is of crucial importance, it will not only help the governments but also, the medical practitioners to know the future trajectory of the spread, which might help them with the best possible treatments, precautionary measures and protections. In this study, the popular autoregressive integrated moving average (ARIMA) will be used to forecast the cumulative number of confirmed, recovered cases, and the number of deaths in Pakistan from COVID-19 spanning June 25, 2020 to July 04, 2020 (10 days ahead forecast).

**Methods:**

To meet the desire objectives, data for this study have been taken from the Ministry of National Health Service of Pakistan’s website from February 27, 2020 to June 24, 2020. Two different ARIMA models will be used to obtain the next 10 days ahead point and 95% interval forecast of the cumulative confirmed cases, recovered cases, and deaths. Statistical software, RStudio, with “forecast”, “ggplot2”, “tseries”, and “seasonal” packages have been used for data analysis.

**Results:**

The forecasted cumulative confirmed cases, recovered, and the number of deaths up to July 04, 2020 are 231239 with a 95% prediction interval of (219648, 242832), 111616 with a prediction interval of (101063, 122168), and 5043 with a 95% prediction interval of (4791, 5295) respectively. Statistical measures i.e. root mean square error (RMSE) and mean absolute error (MAE) are used for model accuracy. It is evident from the analysis results that the ARIMA and seasonal ARIMA model is better than the other time series models in terms of forecasting accuracy and hence recommended to be used for forecasting epidemics like COVID-19.

**Conclusion:**

It is concluded from this study that the forecasting accuracy of ARIMA models in terms of RMSE, and MAE are better than the other time series models, and therefore could be considered a good forecasting tool in forecasting the spread, recoveries, and deaths from the current outbreak of COVID-19. Besides, this study can also help the decision-makers in developing short-term strategies with regards to the current number of disease occurrences until an appropriate medication is developed.

## 1 Introduction

Coronavirus disease (COVID-19), caused by the novel severe acute respiratory syndrome coronavirus 2 (SARS-COV-2) has been declared as a global epidemic by the WHO. The emergence of the novel coronavirus disease (COVID-19) was first reported after a bunch of severe pneumonia cases identified by officials in Wuhan, China in December 2019 [1]. The genetic sequence of which was publicly shared by china on 11 January. Initially, it was thought that the virus has been originated from a seafood market in Wuhan. However, human-to-human contacts have driven its rapid spread with a total of 9129146 confirmed cases, including 473797 deaths across the globe until June 24, 2020 [2]. The most affected countries from this pandemic are the USA, Brazil, Russia, Spain, UK, Italy, France, Germany, China, India, Iran, and Pakistan. The first case of COVID-19 emerged in Pakistan on February 26, 2020, there are 192970 cases with 3904 deaths and 81307 recoveries until June 24, 2020. On March 14, 2020, the Pakistani government, closed all the educational institutes in the country, followed by closing all the shopping malls, partial lockdowns, public holidays in all other government institutes, suspending all public transportation, and directing citizens to stay at home.

COVID-19 has been declared as a global threat by the WHO and asked the international community to take it seriously from time to time. The ability to identify the growth rate at which the epidemic is spreading is very important to fight against it and help in governments’ awareness regarding public planning and policy-making to properly address the consequences of the disease. The key motivation behind the current research work is: to accurately forecast the spread of COVID-19 in Pakistan that could help the Govt officials for better planning to minimize its impact.

So far, several studies have been conducted to predict the spread of the COVID-19 pandemic using various mathematical and statistical models. The ARIMA model has been commonly used in the literature to analyze and predict the spread of the disease. To evaluate the prevalence of the COVID-19, ARIMA (1, 0, 4) was selected as the best ARIMA model, while ARIMA (1, 0, 3) was recommended for the prediction of COVID-19 [3].

The rest of the paper is organized into five sections. The first section includes the introduction as discussed above. The second section consists of the related work providing the relevant studies conducted on the forecasting of COVID-19 using time series models. Section 3 includes the methods and material with the main focus on a data source, model, and methods employed for the analysis of time-series data. Whereas, section 4 consists of the results and discussion. Lastly, the conclusion of the paper is presented in section 5.

## 2 Related work

In this section, scientific research work relevant to this study is presented. Generally, this section includes all the related studies that employ time series models that capture trends and patterns of all the events associated with infectious diseases. Secondly, this section will also focus on the use of such methods that strictly focuses on the prediction of epidemiological variables like cumulative cases, deaths, and recoveries from the current pandemic of COVID-19.

Time series models have been effectively implemented in the literature to forecast infectious diseases. For the prediction of infectious diseases that occur in cyclical patterns such as influenza, similar approaches have been used and are widely published [4]. For example, Song et. al., [5] used the ARIMA model to predict the monthly incidents of influenza in China for 2012. Similarly, in order to predict the mutation of influenza A virus Yin et. al., [6] has proposed a time series prediction model. Zhang et al. [7], proposed a seasonally auto-regressive integrated moving average model to predict seasonal influenza in the USA, UK, and China.

With the emergence of COVID-19, there has been a tremendous rise in the scientific research work conducted regarding the forecasting of COVID-19 and published during the last few months. Roosa, et. al., [8] proposed three different real-time forecasting models for the cumulative number of cases in different provinces of China that were previously suggested to predict infectious disease, for example, SARS, Ebola, influenza and dengue. In a similar study, a Susceptible–Exposed–Infectious–Removed (SEIR) model was trained by Yang et al. [9] on epidemiological and integrated population migration data and combined it with artificial intelligence models to predict COVD-19 in China.

Recently, simple mean-field models were used to assess a quantifiable picture of the COVID-19 pandemic spreading in China, Italy, and France [10]. The analysis results reveal that the simple susceptible-infected-recovered-deaths (SIRD) model has the same kinetic parameter irrespective of the country, while the infection and death rates appear to be more variable.

Similarly, an attempt is being made to forecast C0VID-19 in China from February 5^th^ to February 24^th^, 2020 using the generalized logistic model, the Richards growth model, with quantified uncertainty, and a sub-epidemic wave model [8]. In this article, the total number of confirmed cases of the COVID-19 epidemic in Hubei and other provinces of China as of February 9, 2020 has been predicted. Moreover, gamma distribution has been used to predict COVID-19 in the diamond princes cruise ship, where it is evident from the results that reducing the value of R_0_ will significantly reduce the spread in the ship [11].

Forecasting is the most significant tool that allows us to understand the present scenario and plan for the future in a better possible way. For this purpose, the current study focuses on forecasting of the cumulative confirmed cases, recovered cases, and cumulative deaths from COVID-19 using time series models. With the application of these time series models, the aim is to assess and predict 10 days forecast of the cumulative number of confirmed cases, recovered cases, and deaths in Pakistan as well as to estimate the overall trajectory of the pandemic in the country.

## 3 Materials and methods

### 3.1 Data

**Fig 1:**
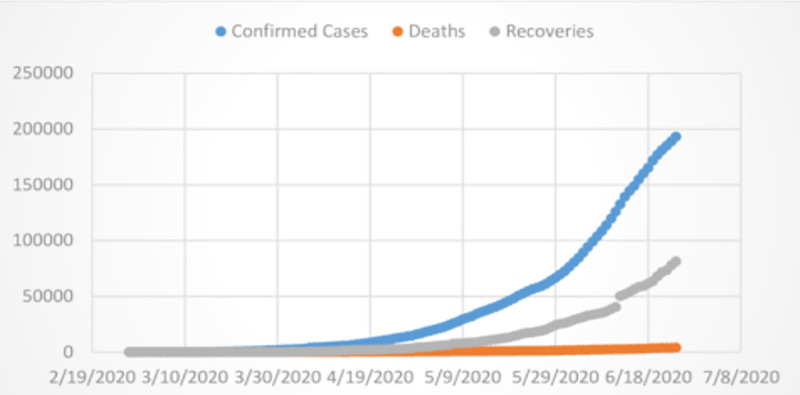
Daily cumulative confirmed, recoveries, and deaths of COVID-19 in Pakistan.

The data for the current study on the number of confirmed cases reported, the number of deaths and recoveries for the COVID-19 were collected from the website of the Ministry of National Health Services, Pakistan [12] during February 26, 2020 and June 24, 2020 and are presented in Fig 1. It can be observed from the Fig 1 that the overall trajectory of the cumulative number of confirmed cases shows an exponential increase, while the growth of cumulative deaths and recoveries are increasing slowly over time. The irregularities and reporting lags affected the time series, so the cumulative curves are more stable and likely yield more reliable estimates. Therefore, the cumulative trajectory of the epidemic in Pakistan was analyzed along with the cumulative aggregate trajectory.

### 3.2 Methods used in this study

Various studies have demonstrated that time series forecasting models focus on the past behavior of a random phenomenon that best captures the underlying trends and patterns. The optimum model is then employed for the prediction of future behavior of the underlying random variable. Over the past few years, there has been tremendous work done on the development of different time series forecasting models for forecasting the pandemics. In this article, several forecasting techniques are implemented such as single exponential smoothing, Holt linear trend method, Holt winter method including the most popular and widely used model that was originally developed for economics applications is the auto-regressive integrated moving average (ARIMA) model [13]. For comparison of these forecasting techniques as demonstrated in several studies in the time series framework (see e.g. Zeng, Q et. al., [14], Zeng and Huang [15], Zeng, Qiang, et al. [16], Zeng, Qiang, et al. [17]), two statistical measures i.e. RMSE and MAE are used to choose the best candidate model for forecasting in the above defined models.

Time series models are used previously for forecasting several epidemics, and infectious diseases including SARS, Ebola, influenza, and dengue [18–22].

#### 3.2.1 The ARIMA Box-Jenkins approach

In this study, the cumulative data were used to forecast the confirmed cases, deaths and recoveries. The reason for using the cumulative cases that the available data is limited and is greatly affected by the variations. It can be seen from Fig 1 that the cumulative number of COVID-19 confirmed cases, recoveries and deaths are likely to show exponential growth overtime, therefore, non-seasonal and seasonal ARIMA model is used to forecast the trend of the current pandemic of COVID-19 in Pakistan. A seasonal ARIMA model can be obtained by adding a seasonal component to the non-seasonal ARIMA model. Based on the values of RMSE and MAE the performance of the ARIMA and SARIMA models which is the combination of the MA and AR term is better than the other exponential smoothing models. The parameters (p, d, q) and (P, D, Q)_m_ of the ARIMA and SARIMA model are selected by the autocorrelation function (ACF) and partial autocorrelation function (PACF). The mathematical structure of the ARIMA model is given in the following equation.

$$x_{t}^{\prime }=a+\gamma_{1}x_{t-1}^{\prime }+\gamma_{2}x_{t-2}^{\prime }+\cdots +\gamma_{p}x_{t-p}^{\prime }+\varphi_{1}\varepsilon_{t-1}+\cdots +\varphi_{q}\varepsilon_{t-q}+\varepsilon_{t}$$ (1)

Where $x_{t}^{\prime }$ is the series that may be differenced more than once. The response variables that appeared on the right-hand side of Eq 1 are the lag values of both the response variable and the error term. Technically, the above-defined model is known as the ARIMA (p, d, q) model, where ‘p’ is the order of the autoregressive term,‘d’ is the degree of differencing the series and ‘q’ is the order of moving average term.

#### 3.2.2 Mean method of forecasting

Mean method of forecasting is very simple and affective especially when the time series do not have very complex behavior. In this method, simple mean of the historical data will be considered as the future forecast. Suppose that the historical values of a time series are denoted by *x*_1,_*x*_2_,…,*x*_*t*_ then the *‘h’* period ahead future forecast values can be written as:

$$F=\bar{x}={(x}_{1}+x_{2}+\cdots +x_{t})/t$$ (2)

#### 3.2.3 Naïve method of forecasting

Naïve method is a simple forecasting technique in which the last period actual values are to be considered as the future forecasted values. The naïve method perform really well in certain circumstances and sometimes perform even better than the other comparable/complicated methods. The naïve method can be symbolized mathematically as follows, i.e.

$$F_{t}=D_{t‐1}$$ (3)

Where F_t_ is the current period forecast that depends upon the previous actual value at time domain D_t-1_.

#### 3.2.4 Seasonal naïve method

Seasonal naïve method of forecast is very much similar to the naïve method, and is very useful when there is high seasonality present in the data. In this method, each forecasted value is equal to the last observed value from the same season of the year. An *‘h’* period ahead forecasted value at time *‘t+h’* is given as:

$${\hat{x}}_{t+h/t}=x_{t+h-m(k+1)}$$ (4)

Where *m =* the seasonal part of the data, and *k* is the integer part of *(h-1)/2*.

#### 3.2.5 Drift method

The drift method of forecasting is nothing but a linear extrapolation. In the first step, a line is drawn between the first and the last point of the data and then this line can be extended to find out the future forecast. One of the advantages of this method over others is that this method is very simple and do not require any complicated mathematical calculation and even can be solved manually. The forecasted value at time *‘t + h’* is given by

$${\hat{x}}_{t+h/t}=x_{t}+\frac{h}{t-1}\sum_{t=2}^{t}(x_{t}-x_{t-1})=x_{t}+h(\frac{x_{t}-x_{1}}{t-1})$$ (5)

#### 3.2.6 Simple Exponential Smoothing (SES) method

The simple exponential smoothing (SES) method is the most common and simplest method of forecasting. This method is a good choice for forecasting the future values when the data have no clear trend or seasonal components. Consider an observed time series *x*_*1*,_ *x*_*2*_, *x*_*3*,_ *…*, *x*_*t*_, then the mathematical structure of the SES takes the following form.

$${\hat{x}}_{t+1}=\alpha x_{t}+(1-\alpha ){\hat{x}}_{t}$$ (6)

Where ${\hat{x}}_{t+1}$ is the forecasted values at time *‘t+1’*, *x*_*t*_ is the current value at time *‘t’*, ${\hat{x}}_{t}$ is the forecasted value at time *‘t’*, and *‘α’* is the smoothing parameter. The forecasted value of *‘x’* at time *‘t+1’* can be obtained by giving weight of ‘α’ to the most recent observation *x*_*t*_ and a weight of ‘1-α’ to the most recent forecast ${\hat{x}}_{t}$.

#### 3.2.7 Holt linear trend method

Holt linear trend method is a two-parameter model, also known as linear exponential smoothing model that can be used for forecasting efficiently the data having a trend component. There are three separate equations for Holt’s method that can be used collectively to produce the final future forecast. The mathematical structure of these equations is given as under.

$${\hat{x}}_{t+h/t}=L_{t}+hd_{t}$$ (7)

$$L_{t}=\partial x_{t}+(1-\partial )(L_{t-1}+d_{t-1})$$ (8)

$$d_{t}=\emptyset^{*}(L_{t}-L_{t-1})+(1-\emptyset^{*})d_{t-1}$$ (9)

Eq 1 represent a forecast equation, Eq 2 is seasonal and Eq 3 represent a trend equation.

Where *L*_*t*_ denotes estimate of the level, *d*_*t*_ represents an estimate of the trend, ∂ is a smoothing parameter for the level and ∅* is a smoothing parameter for the trend. Values of both of these smoothing parameters lies between ‘0’ and ‘1’.

#### 3.2.8 Holt-Winter’s seasonal additive and multiplicative models

The method of holt’s exponential smoothing was extended by [23] and [24] to capture seasonality of the data. If seasonal component of the data is additive then Holt-Winter’s additive method is preferred to obtain good forecasting results, while in case of multiplicative seasonality Holt-Winter’s multiplicative method is preferred. In additive method, the seasonal part of the data is expressed in absolute terms in the same scale of the actual series, and in the level equation the seasonality is adjusted by subtracting it from the observed series. In multiplicative method, the seasonal part of the series is expressed in percentages, and the adjustment is made by dividing through the seasonal component. The mathematical structure of the additive and multiplicative methods is given as:

Holt-Winter’s additive method:

$${\hat{x}}_{t+h/t}=L_{t}+hd_{t}+S_{t+h-m(k+1)}$$ (10)

$$L_{t}=\partial (x_{t}-S_{t-m})+(1-\partial )(L_{t-1}+d_{t-1})$$ (11)

$$d_{t}=\emptyset^{*}(L_{t}-L_{t-1})+(1-\emptyset^{*})d_{t-1}$$ (12)

$$S_{t}=\tau (x_{t}-L_{t-1}-d_{t-1})+(1-\tau )S_{t-m}$$ (13)

Where *‘k’* is the integer part of *(h-1)/m*, which confirms that the projected values of the seasonal indices used for estimation originated from the last year of the sample.

Holt-Winter multiplicative method:

$${\hat{x}}_{t+h/t}=(L_{t}+hd_{t})S_{t+h-m(k+1)}$$ (14)

$$L_{t}=\partial \frac{x_{t}}{S_{t-m}}+(1-\partial )(L_{t-1}+d_{t-1})$$ (15)

$$d_{t}=\emptyset^{*}(L_{t}-L_{t-1})+(1-\emptyset^{*})d_{t-1}$$ (16)

$$S_{t}=\tau \frac{x_{t}}{\left(L_{t-1}+d_{t-1}\right)}+(1-\tau )S_{t-m}$$ (17)

#### 3.2.9 Error Trend Seasonal (ETS) method

Exponential smoothing methods are not only restricted to Holt-Winter’s additive and multiplicative trend. There are nine different combinations of seasonal and trend components are possible. Each method is categorized as a pair of letters *(T*, *S)* that represents the type of ‘trend’ and ‘seasonal component. For illustration purpose (*A*, *M)* is used to denote an ETS model with an additive trend and multiplicative seasonality. Similarly *(A*_*d*_, *N)* is the method with damped trend and no seasonality. Different combinations of the ETS model are presented in the following Table 1.

**Table 1:** Different components of ETS model.

Trend Component	Seasonal Component		
	N (None)	A (Additive)	M (Multiplicative)
**N (None)**	(N,N)	(A,N)	(N,M)
**A (Additive)**	(A,N)	(A,A)	(A,M)
**A**_**d**_ **(Additive damped)**	(A_d_,N)	(A_d_,A)	(A_d_,M)

### 3.3 Forecast evaluation criteria

As one of the important criteria in time series analysis is the forecast evaluation of competing models. To test the robustness and generalizability of different models for the COVID-19 outbreak in Pakistan, two forecasting measures are employed for evaluation in this study. These criteria are root mean square error (RMSE) and mean absolute error (MAE) and their mathematical equations are as follows:

$$MAE=\frac{1}{t}\sum_{i=1}^{t}\left|{\hat{Y}}_{i}-Y_{t}\right|$$ (18)

$$RMSE=\sqrt{\frac{1}{t}\sum_{i=1}^{t}{\left({\hat{Y}}_{i}-Y_{i}\right)}^{2}}$$ (19)

Where *Y*_*i*_ and ${\hat{Y}}_{i}$ represent the original and forecasted values at a given time and *t* associated with the total number of forecasts.

The forecast accuracy of non-seasonal, and seasonal ARIMA, model in terms of RMSE and MAE is better than the other time series models to predict the cumulative number of confirmed cases, recovered cases and deaths from COVID-19 in Pakistan, and hence recommended for forecasting [25]. The proposed model can capture a diversity of trend and seasonal forecasting patterns as well as combinations of the two patterns [26]. These models are opposite to the other modeling approaches to COVID-19 such as using an S-Curve model (logistics curve) that assumes convergence [27]. The mathematical structure of the estimated models for cumulative confirmed cases, recovered cases, and deaths are given in Eqs 1–3.

## 4 Results and discussion

### 4.1 Estimated seasonal ARIMA (p, d, q) (P, D, Q) _m_ model for the cumulative number of confirmed cases

The estimated seasonal ARIMA model is given in Eq 2. This model has one moving average (MA) term, and one seasonal autoregressive component, therefore two model parameters are to be estimated. The estimated value of the parameter that is attached to the non-seasonal part is 0.3636, and the seasonal ARIMA estimated parameter is 0.2305.

$${\hat{Y}}_{t}=0.3636u_{t-1}+0.2305Y_{st-1}$$ (20)

The values for the p, d, and q in the estimated ARIMA model are 0, 2, and 1. Similarly, the values for P, Q, and D are 1, 0, and 0, and the value of m is 7, i.e. 7 days’ seasonality.

### 4.2 Estimated ARIMA (p, d, q) model for cumulative recovered cases and deaths

The estimated ARIMA model for both the cumulative recovered cases and cumulative deaths are given in Eqs 3 and 4, both these models having a single MA term. The estimated parameters of the two models are -0.8189, and -0.5814.

$${\hat{Y}}_{t}=-0.8189u_{t-1}$$ (21)

$${\hat{Y}}_{t}=-0.5814u_{t-1}$$ (22)

The values for p, d, and q in the estimated ARIMA models are 0, 2, and 1, which means that there are zero AR terms, one MA term, and the series is integrated twice to make it stationary.

### 4.3 Cumulative confirmed cases: Forecasts from June 25, 2020 till July 04, 2020

It can be seen from the results produced in Table 2 that the forecasting accuracy in terms of RMSE, and MAE of the Seasonal ARIMA model is better than the other time series models, and therefore is the best candidate model to forecast the next 10 days (25-06-2020 to 04-07-2020) cumulative confirmed cases from COVID-19 in Pakistan.

**Table 2:** Accuracy of different time series models for cumulative confirmed cases.

Method	RMSE	MAE
SES	2499.41	1621.75
Mean	53559.24	42203.94
Naïve	2509.73	1635.32
Seasonal Naïve	17138.84	11261.96
Drift	1903.80	1535.86
Holt’s Linear Trend	422.97	269.17
Holt’s Linear Damped Trend	502.91	305.60
Holt-Winter’s Seasonal Additive	534.92	372.66
Holt-Winter’s Seasonal Multiplicative	975.92	684.93
ETS(A,A,N)	422.97	269.16
**ARIMA(0,2,1)(1,0,0) [7]**	**413.90**	**268.01**

**Fig 2:**
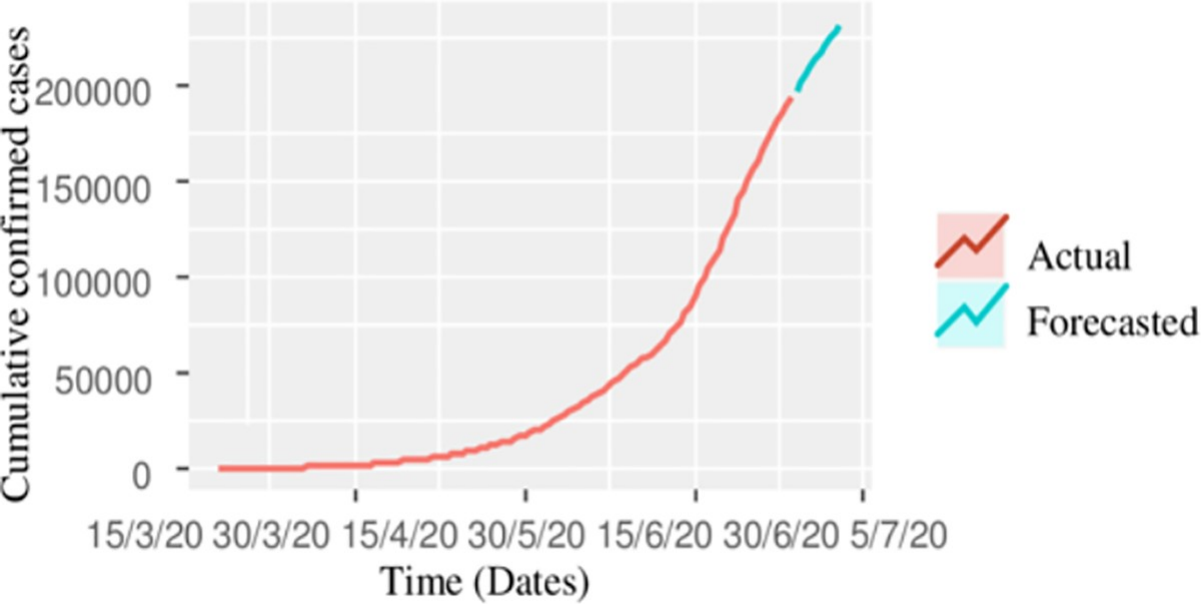
Cumulative actual confirmed cases of COVID-19, together with 10 days ahead forecast.

The point cumulative confirmed forecast cases up to July 04, 2020, are 231239 with a 95% prediction interval of (219648, 242832). The prediction interval means that our point forecast of 231239 lies within this interval, as well as the maximum number of estimated cumulative confirmed cases up to July 04, 2020, are 242832. The actual and forecasted confirmed cases are also shown in Fig 2, it is clear from the figure that the forecasted cumulative confirmed cases are increasing more rapidly and the trajectory is exponential, and yet we still need to flatten the curve.

### 4.4 Forecasts of recovered cases from 25/06/2020 till 04/07/2020

**Fig 3:**
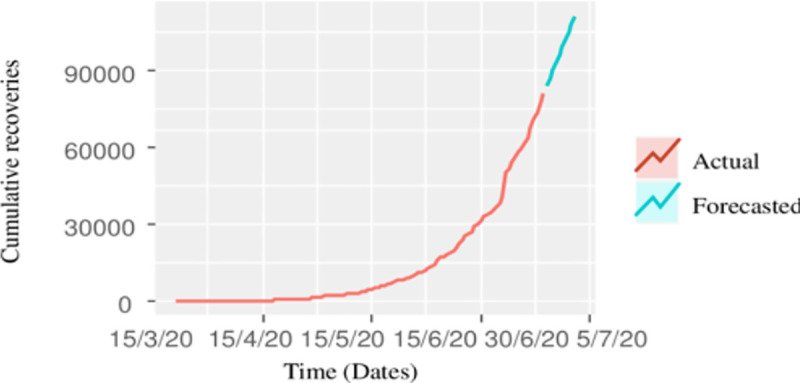
Cumulative actual recovered cases of COVID-19, together with 10 days projection.

In this article, we not only focus on the number of confirmed cases but the number of recovered cases as well. Forecasting the cumulative number of recovered cases are as much important as the cumulative number of confirmed cases. It will help not only the medical professionals but the government officials as well to take further necessary actions in the coming days to combat with COVID-19. The procedure of forecasting the cumulative number of recovered cases is very much similar to that of confirmed cumulative cases. The accuracy of different time series models in terms of statistical measure, i.e. RMSE, and MAE are calculated. It can be seen from Table 3 that the forecasting accuracy of the ARIMA (0, 2, 1) model is better than the other models, and hence the best candidate model to forecast the cumulative recovered cases from COVID-19 in Pakistan. The forecasted cumulative recovered cases up to July 04, 2020, are 1,11,616 with prediction interval of (1,01,063, 1,22,168). The prediction interval means that our point forecast of 1,11,616 lies within this interval, as well as the maximum number of estimated, recovered cases up to June 26, 2020, are 1,22,168. The actual and forecasted recovered cases are also shown in Fig 3, which shows that the recovery rate from COVID-19 is slower as compared to its spread.

**Table 3:** Forecasting accuracy of different time series models for cumulative recovered cases.

Method	RMSE	MAE
SES	1404.38	683.31
Mean	20490.11	15684.08
Naïve	1410.22	689.04
Seasonal Naïve	7480.11	4435.43
Drift	1230.43	757.05
Holt’s Linear Trend	890.40	295.45
Damped Trend	944.86	342.67
Holt-Winter’s Additive	891.31	367.00
Damped Holt-Winter’s Multiplicative	1096.68	511.64
ETS(A,A,N)	890.45	294.40
ARIMA(0,2,1)	**890.30**	**29.20**

### 4.5 Forecasts of cumulative deaths from 25/06/2020 till 04/07/2020

**Fig 4:**
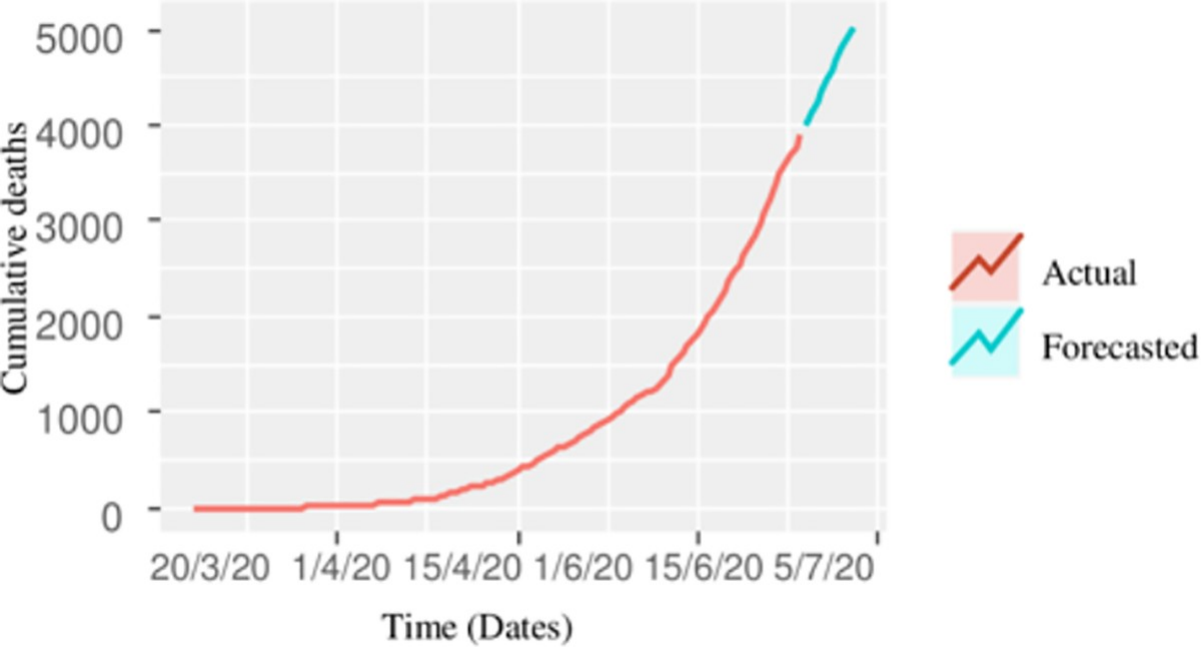
Cumulative actual deaths of COVID-19, together with 10 days projection.

The procedure of forecasting the cumulative deaths is similar to that of cumulative confirmed and recovered cases, the accuracy of different time series models in terms of statistical measure, i.e. RMSE, and MAE is calculated for model assessment. It can be seen from Table 4 that the forecasting accuracy of the ARIMA (0, 2, 1) model is better than the other models, and hence recommended for forecasting. The forecasted cumulative deaths up to July 04, 2020, are 5,043 with a 95% prediction interval of (4,791, 5,295). The prediction interval means that our point forecast of 5,043 lies within this interval, as well as the maximum number of estimated deaths up to July 04, 2020, are 5,295. The actual and forecasted recovered cases are also shown in Fig 4, which shows that the deaths from COVID-19 are much slower as compared to the recoveries.

**Table 4:** Accuracy of different time series models for predicting cumulative deaths.

Method	RMSE	MAE
SES	50.66	32.81
Mean	1059.08	843.06
Naïve	50.87	33.08
Seasonal Naïve	334.86	223.71
Drift	38.64	30.13
Holt’s Linear Trend	12.95	7.25
Damped Trend	14.67	7.99
Holt-Winter’s Additive	12.90	7.54
Damped Holt-Winter’s Multiplicative	1096.68	511.64
ETS(A,A,N)	12.88	7.25
**ARIMA(0,2,1)**	**12.87**	**7.24**

## 5 Conclusion

Based on the forecasted values, the cumulative number of confirmed, recovered, and deaths up to July 04, 2020 will be 2,31,239 with a 95% prediction interval of (2,19,648, 2,42,832), 1,11,616 with prediction interval of (1,01,063, 1,22,168), and 5,043 with 95% prediction interval of (4,791, 5,295) respectively. Based on these forecasted values, the active cumulative confirmed cases from COVID-19 in Pakistan for the next 10 days are estimated to be 1,14,580 (excluding recoveries, and deaths) with a maximum of 1,15,369. As the government of Pakistan, eased lockdown during religious festivals and allowed all the shopping centers to be opened, therefore, resulting in more spread and deaths from COVID-19. If the current policy of the government continued, then in the coming months there will be a disaster, and the actual number of cumulative confirmed cases may be more than the projected, and therefore, our front line medical professionals will fail to deliver, not only our hospitals, but all the places that have been declared as quarantine centers in different cities of Pakistan will be overcrowded. It is time for the government to revise its policy regarding easing the restrictions and opening the businesses to flatten the curve. Otherwise, the situation is going to be worse than the countries that are affected the most from the COVID-19 pandemic.

The results showed the compensations of these algorithms to support strategy/decision-makers in evolving short term policies about the number of disease prevalence. The forecast models will support the government and health staff to be ready for the forthcoming circumstances and take further promptness in healthcare structures. It is worth noting that forecasting is a complex matter, and some tailored models might not be ubiquitous owing to the complex societal and economic circumstances of different nations. The models and predictions proposed in this article do not reflect the local demography, and the real statistics can variate owing to numerous governmental actions like concentration of lockdown, strategy of isolation and health facilities etc. Thus, readers should be careful while interpreting these forecasts.

## 6 Future recommendation

In this research study, an attempt has been made to predict the cumulative number of confirmed cases, deaths, and recoveries of COVID-19 in Pakistan. Here the cumulative data follows an upward or exponential trend, therefore the ARIMA model is used for forecasting. However, ARIMA may perform poorly if the daily deaths, confirmed, and recovered cases follow a nonlinear trend. Similarly, when the data follows a nonlinear trend, then autoregressive conditional heteroscedasticity (ARCH) can be used to forecast the current pandemic of COVID-19. In addition to time series models, machine learning and deep learning tools such as support vector machine (SVM), convolutional neural network (CNN), and recurrent neural network (RNN) can also be used to forecast the COVID-19.

## Supporting information

S1 Data (XLSX)
